# Supplementary material for: Maxillary incisor enamel defects in individuals born with cleft lip/palate
Source: PLoS One. 2020 Dec 28;15(12):e0244506. doi: 10.1371/journal.pone.0244506 (PMC7769421; doi:10.1371/journal.pone.0244506)
Supplement: Attachment — Submitted filename: Rebutal Letter PLOS Juliane.doc [file pone.0244506.s002.doc]

October 12, 2020

TO Editor PLoS One:

Dear Madam/Sir:

We are resubmitting our original work entitled “**Maxillary Incisor Enamel Defects in Individuals Born with Cleft Lip/Palate**” for further appreciation and possible publication in PLoS One. We have devised a new approach to evaluate enamel defects and show for the first time how severity differs depending on the severity of the cleft. We are looking forward to having our work favorably reviewed and considered suitable now for publication.

Sincerely yours,

Alexandre R. Vieira, MS, DDS, PhD

Full Professor of Oral Biology, Pediatric Dentistry, Human Genetics, and Anthropology

Director of Clinical Research

Director of Student Research

School of Dental Medicine

412 Salk Pavilion

335 Sutherland Street

University of Pittsburgh

Pittsburgh, PA 15261

Phone # 412 383-8972

FAX # 412 624-3080

Email: arv11@pitt.edu

Point-by-point answers to the critique are below. All changes in the text are marked in yellow:

Reviewer #1: Thank you for your submission on the important topic. In many areas in the paper there are sentences that are not clear, so some rewriting is required. There are two sets of Tables 1 and 2. I shall make comments and suggestions page by page

Abstract - this appears long, however i do not have a word count
second sentence line 5 - 'has', not 'have'

**RESPONSE: We made the grammatical correction asked.**

3rd last line page 1 - 'individuals with BCLP were 7.85..'

**RESPONSE: We made the requested correction.**

Introduction
3rd para
line 3 - There is, however, a gap
line 4 - the reason 'why' there is..
5th para
'enamel is softer and more porous'

**RESPONSE: We made these corrections as requested.**

The presence of a DDE only potentiates existing increased caries, it doesnt create caries risk i itself - this needs to be made clear

**RESPONSE: The sentence does not state DDE creates risk, but rather that its presence is a risk indicator. The sentence right before mentions “facilitating the development.” We are not making a claim of causal relationship.**

It would be preferential to use maxillary rather than upper

**RESPONSE: We made these changes as requested.**

6th para 'It is' rather than 'Which are'
compare nor compared

**RESPONSE: We changed to “it is” as requested. Since the studies are published, we left the statement that in them it was compared.**

7th para
line 6 - has not have
there is varied use of MMP2 in italics and not

**RESPONSE: We made the correction. MMP2 appears in italic fonts when it refers to the gene and non-italic when it refers to the protein.**

8th para - the verb tenses vary - i prefer past
**RESPONSE: We made the correction to the past tense as suggested.**
M&M
It is unclear how the sample size was determined - was this a convenience sample that was a time limited recruitment process?
para 1 - last word study not report

**RESPONSE: The sample as determined by using all the available material. We added a clarification as suggested. We also made the correction on the style requested.**

para 2
line 4 - surfaces not faces

**RESPONSE: We made the correction as requested.**

stage 1
more details needed for ring flash used
What type of cheek retractors were used? were they sterilised between patients and how?
When was the quality of the image assessed to determine if it needed to be retaken? What metrics were used to asses quality?

**RESPONSE: We added the details assuggested.**

stage 2
Image assessment - after the calibration exercise, were any images assessed by a second examiner to determine inter-examiner agreement and validate continuing correlation with the reference standard?

**RESPONSE: No.**

para 2
'of participants to any of the ..'
inability instead of impossibility

**RESPONSE: We made the corrections as requested.**

Table1 (in M&M) - this isnt the modified DDE index anymore as you have modified it. I suggest creating a name for your index

**RESPONSE: We incorporated the suggestion as stated.**

Stage 3
You mention 'total area' was measured - how was this achieved without some sort of measurement reference in the images? without a reference you cannot measure area, only proportion of area

**RESPONSE: That is what we meant. We clarified the text to address the point raised.**

para 2 - delete 'approximately 10% of the sample' - this is superfluous
**RESPONSE: It was deleted as requested.**

Results
the first paragraph should be moved down one paragraph, it is out of order

**RESPONSE: We made the suggested change.**

Table 1 (results) and Table 2 (results) should be deleted and incorporated into the text (most of the information is there already)

**RESPONSE: We deleted the tables as suggested.**

CLP has already been defined

**RESPONSE: We deleted the definition as suggested.**

Table 3 would benefit by having statistical analysis results added

**RESPONSE: We added the p-values as requested.**

I suggest using the term 'teeth' rather than 'elements' throughout the text

**RESPONSE: We made the requested change.**

in para starting 'As expected' - delete 'as expected' - this is results not discussion

**RESPONSE: It was deleted as suggested.**

In table 4 - no need for % with the numbers as the unit is %SAD

**RESPONSE: We made the correction as suggested.**

in para below Table 4 - fourth line - delete 'counting' and 'to find'. Also, maxillary rather than 'upper'
last sentence before Table 5 - 'between the lateral incisor and canine'

**RESPONSE: We made the suggested corrections.**

Tables 5 and 6 - no need for p= in the table as header is 'p value'

**RESPONSE: We made the corrections as suggested.**

Para after table 5 - 'enamel defects in all thirds' (same for following paragraph)

**RESPONSE: We made the suggested correction.**

Table 6 - where is code 9 data?

**RESPONSE: We added the missing information as resquested.**

Discussion - first para - primary not deciduous

**RESPONSE: We made the correction as requested.**

para 2 - there should be a better word than tactility

**RESPONSE: We changed the word as requested.**

At this stage you should discuss that only 1 examiner/rater was used and the limitations this places on the validity of the results

**RESPONSE: We added the comment in the discussion as requested.**

Para 4 - the sentence starting 'however, the first can reach..' is unclear, especially the reference to 'one third'

**RESPONSE: We rewrote the sentence as suggested.**

para 6- delete 'studies have demonstrated' and primary teeth not deciduous

**RESPONSE: We made the suggested corrections.**

para 8 - sentence starting 'regarding studies on dental...' is unclear and needs rewriting
In the sentence starting one hypothesis - rehabilitation and risk is mentioned - these has no context at present
final sentence - add 'for' after necessary

**RESPONSE: We made the corrections as suggested.**

there is some variability in the format of the references - e.g., some have months and issue numbers, some don’t

**RESPONSE: We removed months as suggested.**

Reviewer #2: - This manuscript describes the results of a cohort cross-sectional study performed on 233 individuals born with cleft lip and/or palate. The authors aimed to evaluate the frequency, location, severity, and extent of developmental enamel defects found in the maxillary incisors as well as understand their relationship with the cleft side. Furthermore, the authors addressed the hypothesis that developmental enamel defects can be influenced by variation in the MMP2 genes (rs9923304). A better understanding of the association between oral clefts and developmental enamel defects will inform dental practitioners in preventing, diagnosing, and treating dental complications that appear to be inevitable consequences of oral clefts.

- As a reviewer with expertise in the field of dental anomalies among individuals with oral clefts, I offer the following perspectives and suggestions that should be considered in the revision of this manuscript.


Introduction:

- Last paragraph: "Thus, the aim of this study was to investigate (the) characteristics of enamel defects and individuals born with oral clefts. " The word (the) is missing.

**RESPONSE: We made the correction requested.**

Methods:

- The study offers a clear description of the study objectives, outcomes, outcome assessment methods, and exclusion criteria. The study describes examiners' calibration and reports the Intraclass Correlation Coefficient.

- First paragraph: "233 individuals born with CLP were evaluated, with (a) mean age of 13.13 years (ranging from 6 to 35 years-old)." The word (a) is missing.

**RESPONSE; We made the correction.**

- Stage 1 – Intraoral photographs: "After (an) initial examination, the surfaces of the teeth were cleaned and dried and the appearance of the enamel was recorded using a digital camera (Canon EOS Rebel T5i, Ohta-ku, Tokyo, Japan), with standard lens (Canon EF 100 mm macro lens) and settings (ISO 6400, speed 1/125 and aperture F/25), always under the same flash (Macro Ring Flash Sigma) and natural lighting conditions. " The word (an) is missing.

**RESPONSE: We made the correction requested.**

- While the authors talk about syndromic/non-syndromic patterns of oral clefts in their introduction, they don't mention the syndromic status of their participants in the methods section.

**RESPONSE: We added the information in the first paragraph of the methods section to address this comment.**

- Furthermore, the authors need to shed the light on the history of surgical and orthodontic treatment among their participants as such treatment may have an effect on the development of enamel defects among individuals with oral clefts.

1. Carpentier, S, Ghijselings, E, Schoenaers, J, Carels, C, Verdonck, A. 2014. Enamel defects on the maxillary premolars in patients with cleft lip and/or palate: a retrospective case-control study. Eur Arch Paediatr Dent. 15(3):159–165.

2. Korolenkova, MV, Starikova, NV, Udalova, NV. 2019. The role of external aetiological factors in dental anomalies in non-syndromic cleft lip and palate patients. Eur Arch Paediatr Dent. 20(2):105–111.

3. Marzouk T, Alves IL, Wong CL, DeLucia L, McKinney CM, Pendleton C, Howe BJ, Marazita ML, Peter TK, Kopycka-Kedzierawski DT, Morrison CS, Malmstrom H, Wang H, Shope ET. Association between Dental Anomalies and Orofacial Clefts: A Meta-analysis. JDR Clin Trans Res. 2020 Oct 8:2380084420964795. DOI: 10.1177/2380084420964795. Epub ahead of print. PMID: 33030085.

**RESPONSE: We added a comment regarding surgical and orthodontic treatment as suggested in the first paragraph. Photographic material is obtained prior to orthodontic treatment and many years after corrective cleft surgery for the lip. The references the reviewer listed also include changes in other teeth not studied by us.**

- The examiner's calibration (Stage 2 – Determination of cleft and DDE phenotypes) is confusing to the reader. According to the study, the initial examination was performed by R.H.W.L and then the study mentions " To eliminate inter-examiner differences, intraoral photographs of all participants were examined by the same evaluator (J.R.L)". What do the authors mean by the word "same"? Who performed the primary evaluation?

**RESPONSE: We edited the text to clarify this concern. R.H.W.L. did all photographic documentation and J.R.L. did all the measurements in the existing photographs.**

- Stage 4 – DNA samples and genotyping: "A single nucleotide polymorphisms (SNP) in the MMP2 gene (rs9923304) was selected, considering disequilibrium linkage and gene structure." Remove the letter (s) from "A single nucleotide polymorphism(s)"

**RESPONSE: We made the correction requested.**

- Stage 5 – Statistical analysis: "In order to verify (the) normal distribution of the numerical variables, the Shapiro-Wilk test was applied, followed by (an) analysis of variance with the Student’s t-test and the Mann-Whitney test, in the cases of normal and non-normal distribution, respectively." The word (the) and (an) are missing.

**RESPONSE: We made the corrections described.**

- As many clinicians may not be familiar with the "cohort cross-sectional study design", it may be useful for the authors to report the limitations of such design. Refer to (Hudson JI, Pope HG Jr, Glynn RJ. The cross-sectional cohort study: an underutilized design. Epidemiology. 2005 May;16(3):355-9. DOI: 10.1097/01.ede.0000158224.50593.e3. PMID: 15824552)

**RESPONSE: We added the limitations as suggested in the discussion section.**

- While the authors reported that the number of teeth and not individuals were used for outcome assessment due to the small sample size available. It should be highlighted that one of the limitations of the present study is the presence of a limited number of participants in the unilateral and bilateral cleft lip groups. (n=19 uCL; n=8 bCL)

**RESPONSE: We added this point as well in the discussion section.**

- The authors should try to offer an explanation of why the means and medians of the percentage of the surface area affected by the defect of the elements inside the cleft palate area and outside the bilateral cleft lip area were zero.

**RESPONSE: We added the requested explanation as a note to the table.**

Results:

- The results section is confusing to the reader who seeks more definitive conclusions.

**RESPONSE: The other reviewer suggested some edits that may help with this concern.**

Discussion

- The discussion section is well written.

- 8th paragraph: "Regarding studies on dental anomalies, Wangsrimongkol et al. (2013) found that the most prevalent missing teeth (was) found in 70.7% of subjects in BCLP group [43]." Replace (was) with (were).

**RESPONSE: We made the correction suggested.**

- While the authors highlighted the significance of genetic factors as a possible cause for the development of enamel defects among individuals with oral clefts, it is important to point out that previous orthodontic and/or surgical treatment can also contribute.

1. Carpentier, S, Ghijselings, E, Schoenaers, J, Carels, C, Verdonck, A. 2014. Enamel defects on the maxillary premolars in patients with cleft lip and/or palate: a retrospective case-control study. Eur Arch Paediatr Dent. 15(3):159–165.

2. Korolenkova, MV, Starikova, NV, Udalova, NV. 2019. The role of external aetiological factors in dental anomalies in non-syndromic cleft lip and palate patients. Eur Arch Paediatr Dent. 20(2):105–111.

3. Marzouk T, Alves IL, Wong CL, DeLucia L, McKinney CM, Pendleton C, Howe BJ, Marazita ML, Peter TK, Kopycka-Kedzierawski DT, Morrison CS, Malmstrom H, Wang H, Shope ET. Association between Dental Anomalies and Orofacial Clefts: A Meta-analysis. JDR Clin Trans Res. 2020 Oct 8:2380084420964795. DOI: 10.1177/2380084420964795. Epub ahead of print. PMID: 33030085.

**RESPONSE: We added a comment in the discussion and these references as suggested.**
