# Supplementary material for: Maxillary incisor enamel defects in individuals born with cleft lip/palate
Source: PLoS One. 2020 Dec 28;15(12):e0244506. doi: 10.1371/journal.pone.0244506 (PMC7769421; doi:10.1371/journal.pone.0244506)
Supplement: Attachment — Submitted filename: Rebutal Letter2 PLOS Juliane.doc [file pone.0244506.s003.doc]

December 9, 2020

TO Editor PLoS One:

Dear Madam/Sir:

We have taken into consideration all the latest review comments and are resubmitting our original work entitled “**Maxillary Incisor Enamel Defects in Individuals Born with Cleft Lip/Palate**” for further appreciation and possible publication in PLoS One. We have devised a new approach to evaluate enamel defects and show for the first time how severity differs depending on the severity of the cleft. We are looking forward to having our work favorably reviewed and considered suitable now for publication.

Sincerely yours,

Alexandre R. Vieira, MS, DDS, PhD

Full Professor of Oral Biology, Pediatric Dentistry, Human Genetics, and Anthropology

Director of Clinical Research

Director of Student Research

School of Dental Medicine

412 Salk Pavilion

335 Sutherland Street

University of Pittsburgh

Pittsburgh, PA 15261

Phone # 412 383-8972

FAX # 412 624-3080

Email: arv11@pitt.edu

Point-by-point answers to the critique are below. All changes in the text are marked in yellow:

Reviewer #1: Thank you for your responses, i believe you have addressed my comments adequately - you will probably find some minor amendments to the written language at final editing

Reviewer #2: Thank you to the authors for revising and resubmitting the manuscript on this important topic. There are a few sentences where rewriting could be beneficial.

Materials and Methods

The last sentence in the first paragraph "Photographic material was obtained prior orthodontic treatment and several years after surgical repair of the lip occurred."

- Please consider rephrasing to "Photographic material was obtained prior [to] orthodontic treatment and several years after [surgical lip repair]" for clarity.

**RESPONSE: We rewrote the text as suggested.**


The last sentence in the second paragraph "The exclusion criteria included labial surfaces of permanent central and lateral incisors not accessible for proper examination (presence of restorations, orthodontic appliances or crowns) or individuals with [bad] quality intraoral photographs."


- Please consider changing [bad] to [low]

**RESPONSE: We made the change as suggested.**


Stage 1 – Intraoral photographs

"Cheeks and lips were retracted using [dental cheek lip retractor mouth opener T-shape], which was sterilized prior [to] each use."

-Please consider changing [dental cheek lip retractor mouth opener T-shape] to [T-Shape intraoral cheek lip retractor]
- Please consider adding [to]

**RESPONSE: We made the suggested changes.**


"When not acceptable because it was not in focused according to the naked eye, the photograph was repeated."

- Please consider rephrasing to [When a photograph was not acceptable because of being out of focus, it was repeated]

**RESPONSE: We made the suggested change.**


Stage 2 – Determination of cleft and DDE phenotypes

Second to the last sentence in the second paragraph

"A new code (9) was added due to the inabibility of observing defects in some teeth, especially those adjacent to the cleft, where the tooth is often distalized, mesialized, ectopic, not erupted or absent"

- Please change [inabibility] to [inability]

**RESPONSE: We made the correction on the typo.**


"Table 1. Classification of defects that includes a modification of the modified DDE index, which [in] the addition of code 9."

- Please consider changing [in] to [includes]

**RESPONSE: We made the correction of the typo.**

"Table 3. Note: *No cases with bilateral cleft lip only had teeth we considered to be outside the cleft [área], since we only evaluated maxillary incisors. Similarly, no cleft palate only cases were affecting maxillary incisors."

- Please consider changing [área] to [area]

**RESPONSE: We made the correction of the typo.**
